# Multi-Dimensional Transcriptome Analysis Reveals Modulation of Cholesterol Metabolism as Highly Integrated Response to Brain Injury

**DOI:** 10.3389/fnins.2021.671249

**Published:** 2021-05-14

**Authors:** Victor Gourain, Olivier Armant, Luisa Lübke, Nicolas Diotel, Sepand Rastegar, Uwe Strähle

**Affiliations:** ^1^Institute of Biological and Chemical Systems-Biological Information Processing (IBCS-BIP), Karlsruhe Institute of Technology (KIT), Karlsruhe, Germany; ^2^UMR 1064 Centre de Recherche en Transplantation en Immunologie, Nantes, France; ^3^PSE-ENV/SRTE/LECO, Institut de Radioprotection et de Sûreté Nucléaire (IRSN), Cadarache, Saint-Paul-Lez-Durance, France; ^4^UMR 1188, Diabète Athérothrombose Thérapies Réunion Océan Indien CYROI, Saint-Denis, France; ^5^COS, University Heidelberg, Heidelberg, Germany

**Keywords:** regenerative neurogenesis, zebrafish, cholesterol, regulation of transcription, bioinformatics analysis

## Abstract

Zebrafish is an attractive model to investigate regeneration of the nervous system. Despite major progress in our understanding of the underlying processes, the transcriptomic changes are largely unknown. We carried out a computational analysis of the transcriptome of the regenerating telencephalon integrating changes in the expression of mRNAs, their splice variants and investigated the putative role of regulatory RNAs in the modulation of these transcriptional changes. Profound changes in the expression of genes and their splice variants engaged in many distinct processes were observed. Differential transcription and splicing are important processes in response to injury of the telencephalon. As exemplified by the coordinated regulation of the cholesterol synthesizing enzymes and transporters, the genome responded to injury of the telencephalon in a multi-tiered manner with distinct and interwoven changes in expression of enzymes, transporters and their regulatory molecules. This coordinated genomic response involved a decrease of the mRNA of the key transcription factor SREBF2, induction of microRNAs (*miR-182*, *miR-155*, *miR-146*, *miR-31*) targeting cholesterol genes, shifts in abundance of splice variants as well as regulation of long non-coding RNAs. Cholesterol metabolism appears to be switched from synthesis to relocation of cholesterol. Based on our *in silico* analyses, this switch involves complementary and synergistic inputs by different regulatory principles. Our studies suggest that adaptation of cholesterol metabolism is a key process involved in regeneration of the injured zebrafish brain.

## Introduction

The capacity of the human brain to regenerate damaged tissue is very limited. In contrast, the central nervous system (CNS) of some fish species has a remarkable ability to regenerate with full restoration of functions (Becker et al., 2004; März et al., 2011; Kaslin et al., 2013; Diotel et al., 2020; Jurisch-Yaksi et al., 2020). For example, deep wounds inflicted by stabbing the brain of the zebrafish with a needle will fully heal. For this (März et al., 2011; Kaslin et al., 2013) and all its other experimental advantages (Kalueff et al., 2014; Marques et al., 2019), the zebrafish has become a powerful model for the analysis of regeneration of the CNS of vertebrates. Although many brain regions show strong cell proliferation and regenerative responses to injury (Kaslin et al., 2017), the telencephalon has emerged as a favored tissue to study regenerative neurogenesis in zebrafish. The everted structure of the teleost telencephalon presents proliferative cell bodies immediately below the skull and the thin tela choroidea at the dorsal surface (Lam et al., 2009; März et al., 2010a; Folgueira et al., 2015; Alunni and Bally-Cuif, 2016). The telencephalic hemispheres are thus easily accessible to wounding and observation without damaging other brain tissues (März et al., 2011; Kizil et al., 2012; Bardehle et al., 2013).

Radial glia cells (RGCs) are the stem cells driving the regenerative response in the zebrafish telencephalon (Pellegrini et al., 2007; Lam et al., 2009; März et al., 2010a). Their cell bodies are scattered at the periventricular surface from the everted dorsal aspects of the medial areas of the telencephalon. Their thin processes span the entire parenchyma. RGCs differentiate into either neuronal cells or, in self-renewing cycles, into more radial glial cells (Than-Trong and Bally-Cuif, 2015; Dray et al., 2021). In response to injury, RGCs undergo mostly symmetric divisions (Barbosa et al., 2015).

Cell death was an immediate reaction to damage of the tissue by 4 hours post lesion (hpl) followed by recruitment of microglia and peripheral immune cells to the lesion (März et al., 2010b; Kroehne et al., 2011; Baumgart et al., 2012; Kyritsis et al., 2012). Edema developed at 24 hpl (Kroehne et al., 2011). Oligodendrocytes and oligodendrocyte precursors accumulate at the site of the injury similar to what is observed in the mouse brain (März et al., 2011; Ghaddar et al., 2021). However, in contrast to the mouse brain, oligodendrocytes or their precursor did not proliferate significantly in the zebrafish (März et al., 2011; Baumgart et al., 2012). By 48 hpl, the RGCs start to divide at a higher rate above the baseline levels characteristic of constitutive neurogenesis (Lam et al., 2009; März et al., 2011; Kizil et al., 2012; Diotel et al., 2013). When only one hemisphere of the telencephalon is injured, this proliferative response as well as gene activation is entirely restricted to the injured hemisphere (März et al., 2011; Rodriguez-Viales et al., 2015). Thus, signals increasing proliferative responses are limited to the injured hemisphere despite the close juxtaposition of the ventricular surfaces in the medial region of the telencephalon. Proliferation of RGCs reaches a peak at 6–8 days after lesion (dpl) and then decreases steadily again reaching basal levels by 10 days (März et al., 2011; Diotel et al., 2020). This proliferation of RGCs can be triggered by inflammatory signals (Kyritsis et al., 2012).

Transcription is a tightly regulated process, where cross-talk between epigenetic marks, transcription factors and their *cis*-regulatory elements orchestrate gene expression. On top of these complex interconnected *cis*- and *trans*-regulatory processes, alternative splicing offers an additional layer to modulate transcriptional responses by increasing the functional diversity of proteins by exon inclusion or exclusion or affecting the stability of mRNAs and proteins (Beyer and Osheim, 1988). Expression levels are further fine-tuned by regulatory RNAs [microRNAs (miRNAs) and long non-coding RNAs (lncRNAs)]. Measuring changes in the repertoire of spliced isoforms and key regulators in relation to differentially expressed gene ontology groups can help deciphering the molecular processes underlying brain regeneration.

Previously, we identified by deep sequencing 252 transcription factor (TF) genes which were up-regulated and 27 TF genes that were down-regulated upon injury (Rodriguez-Viales et al., 2015). The expression pattern of these genes was mapped together with 1,202 constitutively expressed regulators of transcription (Diotel et al., 2015; Rodriguez-Viales et al., 2015). These previous studies focused on the response of transcription factor genes to injury and repair of the telencephalon. Here, we have broadened the analysis of our RNASeq data to all gene ontologies to identify pathways and biological processes that are activated or repressed in response to injury. Besides the expected processes such as neurogenesis and axonal growth, we identified, among many others, genes related to cholesterol metabolism to be differentially expressed in response to injury. This response was multi-tiered and highly coordinated. While mRNAs encoding synthesizing enzymes were down-regulated, transporters were up-regulated. Moreover, transcriptional changes indicated regulation of expression at multiple levels, from the down-regulation of the master TF of cholesterol synthesizing enzymes, *Srebf2*, to the up-regulation of miRNAs with target sequences in cholesterol synthesizing enzymes and *Srebf2* itself. Finally, mRNAs of cholesterol transporters and synthesizing enzymes were differentially spliced suggesting alternative splicing as yet another mechanism for fine-tuning cholesterol metabolism. Our data strongly suggest that modulation of cholesterol metabolism is a key process in brain regeneration in the zebrafish. In addition, our study provides the first comprehensive analyses of basal and injury induced expression of miRNAs and long non-coding RNAs and the shifts in splice patterns in the transcriptome of the regenerating zebrafish telencephalon. We thus report here also valuable resources for follow-up studies.

## Materials and Methods

### RNASeq Data Analysis

RNASeq data were generated as described previously (Rodriguez-Viales et al., 2015). Briefly, one telencephalic hemisphere was injured by inserting a syringe needle as described in detail in Schmidt et al. (2014). RNAs were extracted from uninjured and injured telencephalic hemispheres of the adult zebrafish at 5 dpl. Each telencephalic hemisphere was processed separately. The RNAs were then processed to prepare RNASeq libraries following instructions of the supplier of the reagents (Illumina). Samples were sequenced on an Illumina HiSeq1500. The resulting reads were mapped against the zebrafish reference genome GRCz11 with STAR (Dobin et al., 2013). For reads mapped at multiple loci, only the mapping with the highest quality score was outputted. The purpose was to more accurately quantify the expression of genes. Raw read counts at gene level were computed with HTSeq in union mode (Anders et al., 2015). Expression normalization and differential expression analysis were both carried out with DESeq2 (Love et al., 2014). Aberrant values of expression were flagged and corrected with a generalized linear model with DESeq2. Taken into account the high depth of sequencing, i.e., greater than 200,000,000 on average per sample, a gene was considered as expressed with an average normalized level of expression across all samples greater than 100. Differences in expression between control and injured telencephalic hemispheres were assessed also with DESeq2. The *p*-values of the Wald tests were adjusted with the Bonferroni method. A threshold of 0.05 was applied on adjusted *p*-value (adjp) to identify significant changes in expression in response to injury. No threshold was applied on fold change (FC) to exhaustively identify differentially expressed genes.

lncRNA genes transcribed in the adult zebrafish telencephalon were identified based on the tag “biotype” extracted from the annotation of the zebrafish reference genome. The biotype of RNAs was predicted with the alignment of genomic sequences against non-coding sequence of the RFAM database (Kalvari et al., 2018) with BLASTN (Altschul et al., 1990). The resulting alignments were then filtered applying thresholds on e-values and refined with a co-variance model as described on the Ensembl website^1^. The annotation of the neighboring genes directly upstream and downstream, with no threshold of distance, was carried out with the R packages GenomicRanges (Lawrence et al., 2013).

For the functional annotation of the zebrafish genome, the latest gene ontology terms (Ashburner et al., 2000), signaling pathways (Kanehisa and Goto, 2000), and metabolism pathways (Fabregat et al., 2018) were retrieved from their respective database. The enrichment was then tested with the one-tailed exact Fisher test, as previously published (Cato et al., 2017). The Fisher *p*-values were corrected with the False Discovery Rate (FDR) method as previously published (Cato et al., 2017). A threshold of 0.05 was applied on corrected *p*-values to identify significantly enriched biological functions.

The two binding motif of SREBF2 identified in vertebrates were retrieved from the database JASPAR 2018 (Khan et al., 2018) and were mapped, with HOMER (Heinz et al., 2010), in the promoter sequence of all genes with significant variation in the level of transcripts in response to injury. The promoter region was defined as 1-kb upstream of the transcription start site, provided by the annotation of the zebrafish reference genome. For the mapping of sterol regulatory element (SRE) with HOMER, a background set was created with the same number of sequence, i.e., 4,989, randomly extracted from the zebrafish reference genome GRCz11. The background sequences were also of the same size, i.e., 1 kb. Both forward and reverse strands were analyzed.

To investigate alternative splicing of polyadenylated RNAs, transcripts synthesized in the adult zebrafish telencephalon were first *de novo* reconstructed from mapped RNASeq reads with STAR. The mapping of the reads at splicing junction was refined with a second pass taken into account splicing junctions identified in both control and injured RNASeq samples. From the mapped reads, transcripts were *de novo* reconstructed, with Leafcutter (Li et al., 2018), with no limits in the number of introns per transcript and novel splice junctions supported by a minimum of 20 split reads. For each transcript differential splicing between control and injured telencephalic hemisphere was assessed with Leafcutter as well. The *p*-values were corrected with the FDR method as recommended. Significant alternative splicing of transcripts in response to injury were identified with two parameters: 1. applying a threshold of 0.05 on adjp, 2. the corresponding splicing junction was covered by at least 20 mapped reads. Results were visualized with the genome browser IGV (Thorvaldsdóttir et al., 2013) and transcript isoforms were manually reconstructed. The sequence of spliced exons was retrieved from Ensembl (Yates et al., 2020) and the corresponding protein domains were identified with the software InterPro (Mitchell et al., 2019) relying on annotation of protein domains present in the database UniProt (UniProt Consortium, 2019).

### Sequencing of Small RNAs and MicroRNA Analysis

After an identical preparation of RNAs as described above and in Rodriguez-Viales et al. (2015) small RNA libraries were prepared from 1 μg of total RNAs with the Small RNA Library Preparation kit (Illumina) following the manufacturer’s protocol. Three libraries for control and injured telencephalic hemispheres were sequenced with a HiSeq1500 (Illumina). The adaptor sequence (Illumina) was trimmed from raw reads with Cutadapt (Martin, 2011) for a final insert size of 21, 22, or 23 nucleotides.

Passing all quality controls carried out with FASTX toolkit^2^, reads were mapped against the zebrafish reference genome GRCz11 with STAR (Dobin et al., 2013). No soft-clippings were allowed, only one mismatch was allowed and only mappings with a quality of 30 (Phred score) were outputted. Raw read counts were computed with HTSeq (Anders et al., 2015) in union mode and with an annotation file including all known miRNA loci in the annotation of the zebrafish reference genome GRCz11, as recommended by the ENCODE project (ENCODE Project Consortium, 2012). Expression normalization and differential expression analysis were both carried out with DESeq2 (Love et al., 2014), as described above. A threshold of 0.05 was applied on adjp to identify significant changes in steady state levels of miRNAs upon injury. To identify strong changes in levels of miRNAs upon injury, thresholds of 0.25 and 2 were applied on FC. A miRNA was considered as expressed with an average normalized level of expression across all samples greater than 10. Predicted target mRNAs, specific for the zebrafish, were retrieved from the database TargetScanFish (Ulitsky et al., 2012). No filters were applied on the tissue where the miRNAs were originally expressed.

### Preparation of Biological Samples and qRT-PCR

Injury was inflicted to the telencephalon as described previously (März et al., 2011). For qRT-PCR, total RNA was isolated from injured and uninjured telencephalic hemispheres using Trizol (Life Technology). First strand cDNA was synthesized from 1 μg of total RNA with the Maxima First Strand cDNA kit (Thermo Fisher Scientific) and according to the manufacturer’s protocol. qRT-PCR was carried out with a StepOnePlus Real-time qRT-PCR system (Applied Biosystems) and SYBR Green I fluorescent dye (Promega). Expression levels of genes were normalized to β-actin expression and the relative expression levels were calculated using the 2-ΔΔCT method. Real-time qRT-PCR was carried out in triplicates of independently prepared samples and repeated once. Differences in relative expression between control and injured telencephalic hemispheres were tested with the one-tailed *t*-test. The sequence of the primers is provided in Supplementary Table 10.

## Results

### Injury-Induced Changes in Steady State Levels of Polyadenylated RNAs in the Telencephalon

**FIGURE 1:**
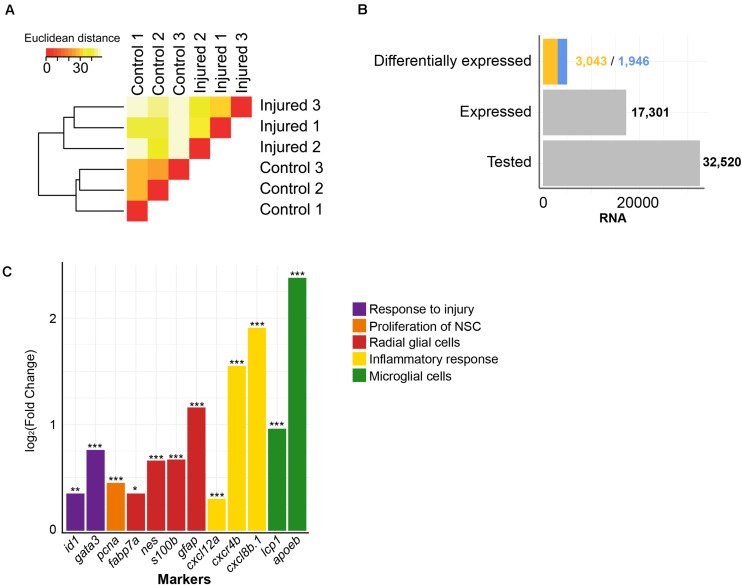
Injury-induced changes in level of polyadenylated RNAs. **(A)** Similarities between transcriptome of RNASeq samples were assessed by hierarchical clustering on the Euclidean distances computed between samples. The RNASeq samples were consistently grouped in their condition, control and injured. **(B)** RNAs were quantified for all annotated genes in the zebrafish reference genome GRCz11 (“Tested,” *n* = 32,520), genes expressed in the adult zebrafish telencephalon (“Expressed,” *n* = 17,301) or genes differentially expressed upon injury (“Differentially expressed”). The blue color depicts increased levels of transcript after injury (*n* = 1,946) and the yellow color decreased levels of transcript after injury (*n* = 3,043). **(C)** The sensitivity of the RNASeq analysis was further evaluated with significant changes in level of RNAs of relevant markers for regenerative neurogenesis. These markers were, selected based on literature, were grouped according to respective biological functions relative to the repair of damaged adult zebrafish telencephalon (color code). *adjp = 0.05, **adjp < 10^– 02^, ***adjp < 10^– 04^.

To get a comprehensive picture of the transcriptional changes caused by injury of the adult brain, we re-analyzed previously established RNASeq data (Rodriguez-Viales et al., 2015). The sequenced cDNA was derived from polyadenylated RNA isolated from injured telencephala of the adult zebrafish at 5 dpl, with the contralateral hemisphere as uninjured control (Rodriguez-Viales et al., 2015). We analyzed in total approximately 600,000,000 reads from injured telencephalic hemispheres and an equal number of reads from uninjured control hemispheres. The RNASeq samples from the three biological replicates of each condition were consistent as assessed by hierarchical clustering (Figure 1A). A total of 32,520 genes annotated in the zebrafish reference genome GRCz11 were tested and 17,301 were expressed in the adult zebrafish telencephalon (Figure 1B). The analysis of differential expression revealed 1,946 and 3,043 genes with significantly up- or down- regulated expression, respectively (adjusted *p*-value (adjp) < 0.05) (Figure 1B and Supplementary Table 1), relative to the transcriptome of the uninjured hemisphere.

To assess the sensitivity of our analysis, we selected genes known from previous studies to be altered in their level of expression by injury of the telencephalon (Figure 1C). The transcription factor *gata3* is a gene which responds to injury of the telencephalon very rapidly (Kizil et al., 2012), and is followed by the transcription regulator *id1* (Rodriguez-Viales et al., 2015). In agreement, the level of transcripts coding for Gata3 and Id1 were significantly increased upon injury (Fold Change (FC) = 1.70 and 1.30, respectively; adjp < 10^–04^ and adjp < 10^–02^, respectively). Similarly, transcripts coding for proliferation cell nuclear antigen (PCNA), a marker of dividing cells (Romero-Alemán et al., 2004), were elevated after injury (FC = 1.37; adjp < 10^–04^), as well as mRNAs of the RGC-specific genes *fabp7a*, *nestin*, *s100b*, *glial fibrillary acidic protein* (*gfap*) (FC = 1.27, 1.58, 1.59, and 2.23, respectively; adjp < 0.05, < 10^–05^, < 10^–05^ and < 10^–24^, respectively) (Lam et al., 2009; Moullé et al., 2012). We also observed that mRNAs encoding Apoeb and Lcp1, markers for microglia (Nakai et al., 1996), were up-regulated upon injury (FC = 5.21 and 1.95, respectively; adjp < 10^–67^ and < 10^–06^, respectively) as were mRNAs of the cytokines *cxcl8b.1* and *cxcl12a* (FC = 2.93 and 1.23, respectively; adjp < 10^–35^ and < 10^–03^, respectively) and the cytokine receptor *cxcr4b* (FC = 3.73; adjp < 10^–02^). The increased expression of these genes coding for cytokines and cytokine receptors reflects the activation of an inflammatory response by injury (Kyritsis et al., 2012). Taken together, all assessed genes whose expression levels are known to be regulated by injury were verified in our transcriptome analysis (Figure 1C). These results show that we detected variation of transcript levels in response to telencephalon injury with high sensitivity.

### Gene Ontology Analysis

Next, we assessed the enrichment of specific ontologies among regulated genes to obtain information on the biological processes that are linked to the repair of the injured telencephalon. Three sources of data provided information on biological functions [Gene Ontology (GO), (Ashburner et al., 2000)], signaling pathways (Kyoto Encyclopedia of Genes and Genomes (KEGG), Kanehisa and Goto, 2000) and metabolic pathways (Reactome, Fabregat et al., 2018). A total of 192 GO terms, 34 KEGG pathways and 295 Reactome enzymatic reactions were significantly enriched among the genes with variation in level of transcripts upon injury (adjp < 0.05) (Supplementary Table 2).

**FIGURE 2:**
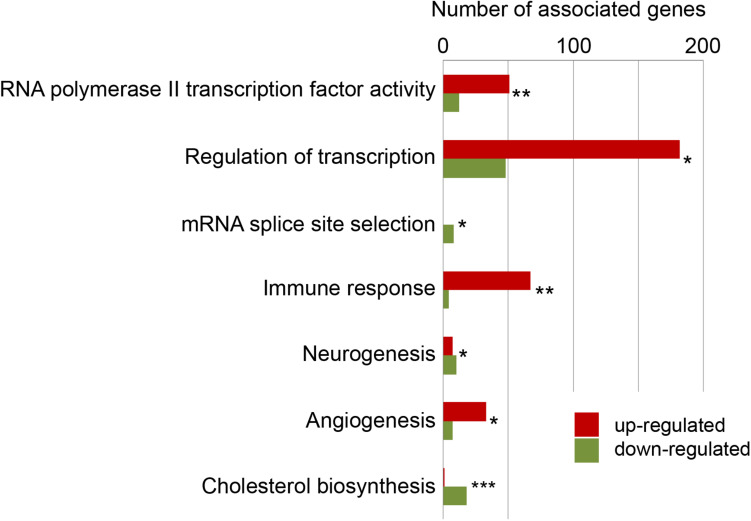
Selected enriched biological functions. To gain information about functions of genes with significant changes in their levels of transcript, the enrichment of ontologies (GO terms), signaling pathways (KEGG pathways) and metabolism pathways (Reactome pathways) was tested and up-regulated genes (red) and down-regulated genes (green) were detailed. *adjp = 0.05, **adjp < 0.01, ***adjp < 0.001 (For complete list see Supplementary Table 2).

A major response was generic transcription regulation comprising, among others, the GO terms “RNA polymerase II transcription factor activity” (associated with 63 differentially expressed genes; adjp < 10^–02^) and “Regulation of transcription” (represented by 230 differentially expressed genes; adjp < 10^–21^). This broad response of the transcription regulator genes is consistent with previous reports (Diotel et al., 2015; Rodriguez-Viales et al., 2015) and reflects the large-scale, injury inflicted changes of the transcriptome. Other GO terms–expected from the response of the genome–were “neurogenesis,” “angiogenesis,” “immune response” (Figure 2). Beside these expected functions among the regulated genes, we detected a large number of distinct enriched gene ontology terms including “mRNA splice site selection” (adjp = 0.025) and “cholesterol metabolism” (adjp < 10^–04^).

### Expression of Enzymes and Transporters of Cholesterol Metabolism Are Co-ordinately Regulated in Response to Injury

**FIGURE 3:**
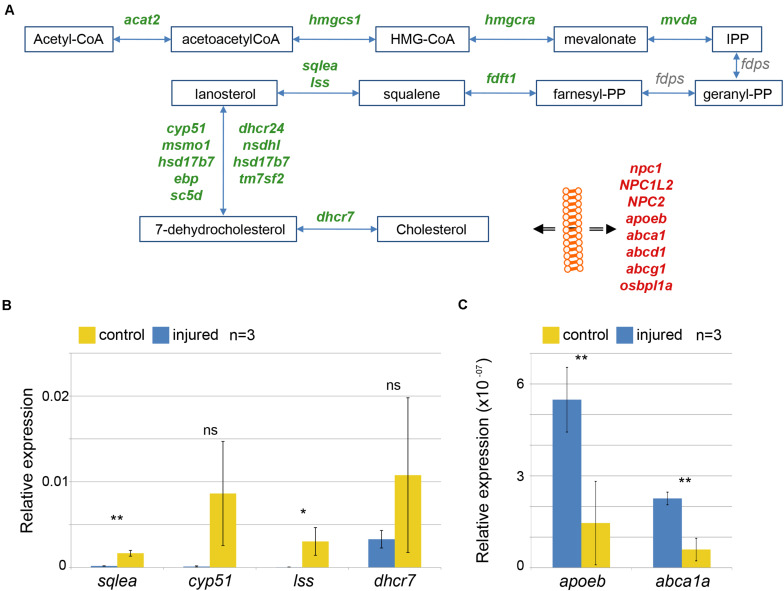
Alteration in cholesterol metabolism in response to brain injury. **(A)** Increases in level of transcripts coding for cholesterol synthesizing enzymes (green) and decreased level of transcripts coding for transporter involved in ferrying cholesterol through the body and across membrane (red) were identified. Products and substrates are represented in blue boxes and enzymatic reactions by blue arrows. The double black arrow represents flow across membrane. **(B,C)** Changes in levels of mRNA were validated comparing the quantification by qRT-PCR of mRNAs encoding three selected enzymes synthesizing cholesterol **(B)** and two transporters **(C)** in three independent control (yellow) and injured (blue) telencephalic hemispheres (*n* = 3). *p-value = 0.05, **p-value < 10^– 03^.

There is no data available about a role of cholesterol in the regeneration of the zebrafish brain. We focused our analysis on genes linked to cholesterol metabolism. Cholesterol is a component of plasma membranes and is highly abundant in myelin sheaths (Björkhem and Meaney, 2004). The synthesis of cholesterol from acetyl-CoA is a multi-step process involving a large number of distinct enzymes (Sharpe and Brown, 2013). We found that, after injury, levels of transcripts coding for all but one of the enzymes involved in cholesterol synthesis were decreased (Figure 3A and Supplementary Table 3). Cholesterol synthesis is repressed by availability of external cholesterol (Eberlé et al., 2004). Besides synthesis, transport of cholesterol is an important process that contributes to the regulation of cholesterol levels. Upon brain injury, levels of mRNAs coding for 8 cholesterol transporters were significantly increased, i.e. *npc1*, *NPC1L2*, *NPC2*, *apoeb*, *abca1*, a*bcd1*, *abcg1*, *osbpl1a* (Figure 3A and Supplementary Table 3). Taken together, our results indicate that cholesterol transport is increased while its synthesis is turned down.

Although our RNASeq transcriptome analysis detects genes responsive to injury with a high sensitivity and fidelity, we wished to confirm these findings on the cholesterol pathway with an independent method and with different sample preparations. To this end, we carried out a qRT-PCR analysis with a number of selected genes (Figures 3B,C). The metabolic enzymes *sqlea*, *cyp51*, *lss* and *dhcr7* yielded lower signals relative to the uninjured control (Figure 3B) as expected from the transcriptome analysis. Similarly, we detected increases of transporter cDNAs encoding *apoeb* and *abca1a* in the injured sample relative to uninjured control cDNA (Figure 3C). These qRT-PCR results verify our transcriptome analysis and support the hypothesis that cholesterol metabolism is modulated after telencephalon injury. Taken together, this response of the transcriptome suggests that injury results in an increase of available cholesterol, presumably as a result of release from damaged and dying cells.

### Expression of the Master Regulator of Cholesterol Synthesizing Enzymes Srebf2 Is Reduced Upon Injury

Basic helix loop helix SREBF transcription factors regulate expression of cholesterol synthesizing enzymes in mammals (Wang et al., 1994; Eberlé et al., 2004). We thus explored expression of *srebf*. In the zebrafish genome as in that of mammals, two paralogous genes encode the Srebf1 and Srebf2 proteins, and both are expressed ubiquitously in the adult zebrafish telencephalon (AGETAZ database; Diotel et al., 2015). The level of transcripts coding for Srebf2 was significantly lower (FC = 0.63; adjp < 10^–09^) in the transcriptome of the injured zebrafish telencephalon, consistent with the observed lower expression of Srebf2-targeted genes encoding cholesterol synthesizing enzymes. These data suggest that Srebf2 might be the main regulator of cholesterol synthesis in zebrafish as in mammals (Sharpe and Brown, 2013).

**FIGURE 4:**
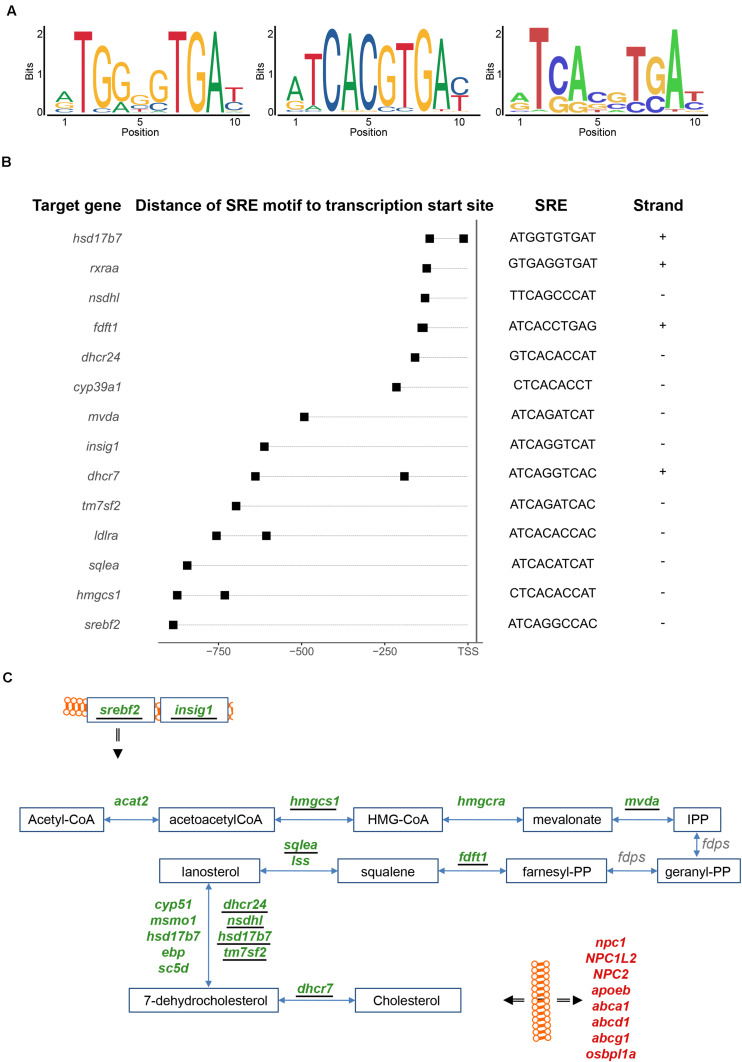
Sterol Regulatory Element (SRE) motif analysis. **(A)** Two mammalian SRE motifs were retrieved from the literature (left and middle panels). From the mapping of these two consensus sequences one SRE motif derived in the zebrafish genome (right panel). **(B)** The SRE motifs were mapped in the promoter of genes involved in cholesterol metabolism. The promoter sequence was defined from 1 kb upstream of the transcription start site and the SRE motif were mapped in both forward (+) and reverse (–) strands. **(C)** Genes harboring a SRE motif in their 1-kb promoter (underlined) were identified in the cholesterol synthesis pathway, including genes coding for two upstream regulators (*srebf2* and *insig1*). For further details see also legend to Figure 3A.

We analyzed thus next the promoters of the genes differentially expressed after injury for potential enrichment of Srebf2 binding sites. Srebf2 interacts with a short target sequence, the Sterol Regulatory Element (SRE), in the promoter region of responsive genes (Sharpe and Brown, 2013). We analyzed whether the two related consensus sequences of mammalian SRE motifs listed in JASPAR (Figure 4A; left and middle panel) were present in the 1-kilobase (kb) promoter sequence of the genes with significant variations in level of transcripts after injury. As a control, we created a set of background 1-kb sequences obtained from randomly chosen loci.

In total, 1,145 genes with changes in expression levels upon injury harbor homologies of a SRE motif in the 1-kb promoter region. Relative to the control, this represents a significant enrichment with positive log odds scores and after correction for GC content and repeat of k-mers (Supplementary Table 6). Moreover, the GO term “cholesterol biosynthetic process” is enriched among these genes carrying a SRE motif (adjp < 0.05) (Supplementary Table 9). By additionally mining the list of SRE harboring genes manually, in total nine genes coding for enzymes involved in the synthesis of cholesterol: *hmgcs1*, *mvda*, *fdft1*, *sqlea*, *tm7sf2*, *nsdhl*, *dhscr24*, *hsd17b7*, and *dhcr7* (Figure 4B and Supplementary Table 4) were found with SRE motifs in the 1-kb promoter region. These results partially overlap with SRE motifs mapped in the promoters of the human and mouse orthologous genes (Sharpe and Brown, 2013; Supplementary Table 4). SRE motifs were also identified in the promoter region of two key regulators of the cholesterol metabolism, *srebf2* itself and *insig1*, a post-translational regulator of Srebf2 (Dong et al., 2012) (Figure 4C). The presence of a SRE binding site in the promoter of Srebf2 suggests an auto-regulatory feedback-loop of *srebf2*. The SRE motifs were also identified in the promoter of other differentially expressed genes involved in cholesterol metabolism (Figure 4B). For example, low-density lipoprotein (LDL) receptor a (ldlra), the alpha sub-unit of the retinoic X acid receptor (*rxraa*) (Repa et al., 2000) and cytochrome P450 family 39 subfamily A polypeptide 1 (*cyp39a1*) (Li-Hawkins et al., 2000), all involved in cholesterol metabolism, were detected as potential Srebf2 transcriptional targets. From the homology scores in the zebrafish genome (Figure 4A; left and middle panel) (Khan et al., 2018), a putative zebrafish Srebf2 sequence was derived (Figure 4A; right panel). The *in silico* predicted sequence is similar to the SREBF2 binding sequence identified in human genes by Selex (Jolma et al., 2013) rather than the Chromatin Immuno-Precipitation (ChIP) followed by Sequencing (ENCODE Project Consortium, 2012) derived consensus sequence (Figure 4A; middle panel).

Taken together, this significant enrichment of SRE motifs in cholesterol biosynthetic genes supports the notion that Srebf2 is also a regulator of the expression of these genes in the zebrafish genome.

### miRNAs That Target Cholesterol Genes Are Increased Upon Injury

**FIGURE 5:**
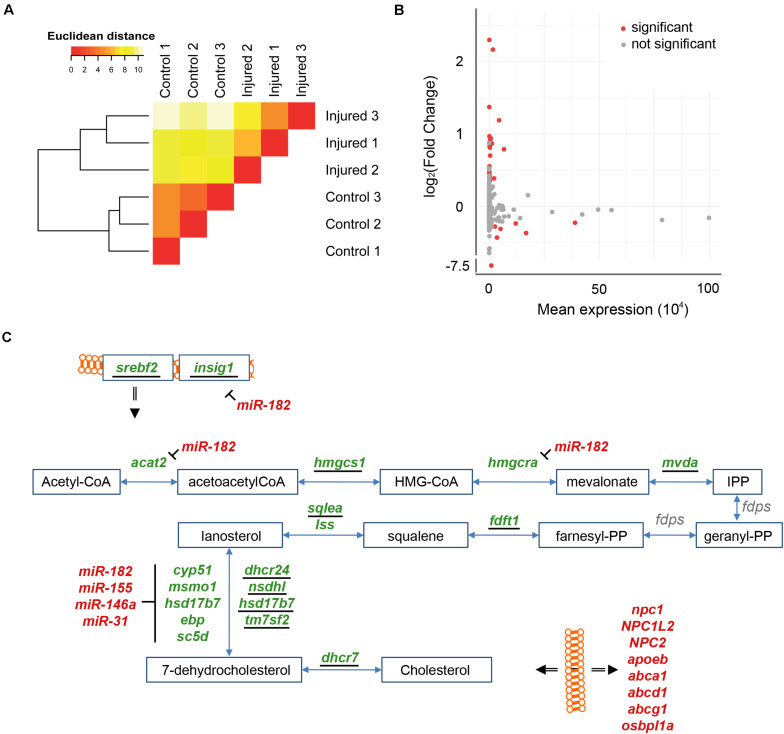
Injury-induced changes in levels of miRNAs. **(A)** The consistency of small RNASeq samples was tested by hierarchical clustering on Euclidean distances as for the RNASeq samples (see Figure 1A). The small RNASeq samples were consistently grouped into their respective condition, control or injured. **(B)** Changes in level of miRNAs were assessed comparing injured and uninjured telencephalic hemispheres. Significant differences in level of miRNA were tested (red; adjp < 0.05). **(C)** Targets of *miR-182*, *miR155*, *miR-146a*, and *miR-31* were identified in the cholesterol synthesis pathway. For further details see also legend to Figures 3A, 4C.

miRNAs are well established negative regulators of coordinated gene programs (Bartel, 2004). The changes in expression of miRNAs were thus investigated by small RNASeq in the injured telencephalic hemisphere in comparison to the uninjured hemisphere. Computation of Euclidean distances and hierarchical clustering between small RNASeq samples grouped the samples according to their respective experimental condition (Figure 5A). A total of 184 miRNAs annotated in the zebrafish reference genome (GRCz11) were detected in the transcriptome of the adult zebrafish telencephalon. The analysis of differential miRNA expression, identified 31 miRNAs regulated at least two fold after injury (adjp < 0.05). Among these, the level of 22 miRNAs increased upon injury while the level of 9 miRNA decreased (Figure 5B and Supplementary Table 7). For further analysis, we focused on the five miRNAs with the strongest variation in their level in response to injury. The level of four miRNAs increased in response to injury: *miR-31* (FC = 4.92; adjp < 10^–64^), *miR-146a* (FC = 4.50; adjp < 10^–62^), *miR-155* (FC = 2.58; adjp < 10^–09^) and *miR-182* (FC = 2.28; adjp < 10^–02^). The level of *miR-26b*, decreased after injury (FC = 0.0050; adjp < 10^–246^). None of these five miRNAs were previously shown to be involved in the regulation of constitutive or regenerative neurogenesis.

We next assessed potential mRNA targets of these five miRNAs by screening for the presence of the seed sequence in the 3′UTR of differentially expressed mRNAs. Interestingly, we found the three miRNAs *miR-31*, *miR-146a*, and *miR-155* target them RNAs of five down-regulated genes coding for enzymes of the synthesis of 7-dehydrocholesterol: *ebp*, *cyp51*, *sc5d*, *hsdl7d7*, and *msmo1* (Figure 5C). In addition, the mRNAs encoding Insig1 (FC = 0.43; adjp < 10^–23^), Acat2 (FC = 0.75; adjp < 10^–06^), Dhcr24 (FC = 0.57; adjp < 10^–05^), Sc5d (FC = 0.66; adjp < 10^–03^) and Hmgcra (FC = 0.54; adjp < 10^–12^) were predicted targets of *miR-182* (Figure 5C). Acat2, Dhcr24, Hmgcra, and Sc5d are enzymes participating in the synthesis of cholesterol (Sharpe and Brown, 2013) and Insig1 is a co-factor of Srebf2. Taken together, these data strongly suggest that, in addition to the transcriptional regulation via SREBF2, several miRNAs contribute to the adaptation of the cholesterol metabolism to the altered physiological needs of the injured telencephalon.

### Injury-Induced Changes in Levels of Polyadenylated Long Non-coding RNAs

The vast majority of the known lncRNAs are polyadenylated (Dykes and Emanueli, 2017). Their expression levels can thus be extracted from our RNASeq data. After injury of the adult zebrafish telencephalon, we detected significant changes in the levels of 149 lncRNAs (77 increased and 72 decreased) (Supplementary Table 1). As the functional annotation of lncRNAs is still poor, we scored the putative target protein-coding genes next to the loci encoding lncRNAs, and carried out functional annotation enrichment on these nearby protein-coding genes.

**FIGURE 6:**
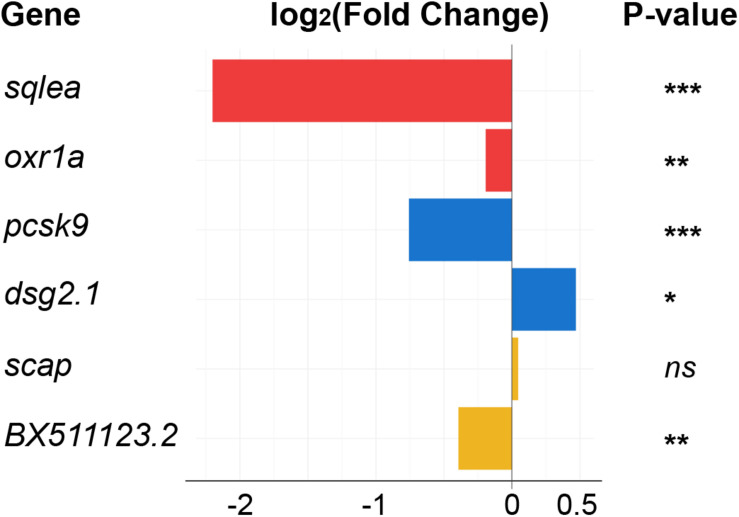
Differentially expressed lncRNAs selected for their association with cholesterol synthesis or transport. LncRNAs annotated in the zebrafish genome, and with significant changes in their respective levels upon injury, were localized in the direct vicinity of genes encoding cholesterol-related proteins. Color indicates pairs of coding and non-coding RNAs. *adjp = 0.05, **adjp < 10^– 02^, ***adjp < 10^– 04^, ns: not significant. See legend of Figure 7B for the position of the genes in the cholesterol pathway.

Several lncRNAs with changed levels in the regenerating telencephalon were identified directly upstream or downstream of cholesterol-related protein-coding genes (Figure 6). The level of both *oxr1a* lncRNAs and its potential downstream target *sqlea*, known to convert squalene to lanosterol during cholesterol synthesis (Sharpe and Brown, 2013), significantly increased upon injury (Figure 3A). Other examples of potential lncRNA transcriptional target include *pcsk9* and the lncRNA, *dsg2.1* which were down and up-regulated, respectively. Pcsk9 is known to regulate cholesterol homeostasis (Poirier et al., 2008). Finally, although no significant change in level was observed for mRNAs coding for *scap*, the level of surrounding lncRNA *BX511123.2* significantly changed in response to injury (Figure 6). Scap is a chaperone of Sreb transcription factors and forms a retention complex in the membrane of the endoplasmic reticulum (ER) (Lee et al., 2020).

Although a regulatory role of any of the lncRNAs has not been established by functional experiments, our data support the hypothesis that lncRNAs are involved in orchestrating the response of the genome to injury of the telencephalon and that they may also more specifically contribute to the regulation of cholesterol metabolism.

### Alternative Splicing of RNAs in Response to Injury of the Telencephalon Affects Cholesterol Metabolizing Enzymes and Transporters

Alternative splicing is a post-transcriptional modification of RNAs that increases the functional diversity of proteins by exon inclusion or exclusion or affects the stability of mRNAs and proteins (Beyer and Osheim, 1988). The enriched gene ontology terms among regulated genes included “mRNA splice site selection” (adjp < 0.05) suggesting that injury may alter the pattern of splicing of mRNAs (Supplementary Table 2). Alternative splicing events were identified by comparing *de novo* reconstructed transcripts present in uninjured and injured telencephalic hemispheres taking the annotation of the zebrafish reference genome (GRCz11) into account. FC and adjp were computed for each alternative splicing event. In total, 4,610 alternatively spliced variants were detected in response to injury (adjp < 0.05), affecting transcripts synthesized from 1,309 genes. Change of ratio of transcript isoforms was the most recurrent difference between uninjured and injured telencephalic hemispheres. We also identified novel isoforms of RNAs specific for the adult zebrafish telencephalon and which had not yet been annotated in the zebrafish reference genome (GRCz11) (Supplementary Table 8). Thus, brain injury results in a large change of splicing patterns.

These results were further refined according to the biological functions of genes from which alternatively spliced RNAs were synthesized. *Mbpa* (FC = 2.6; adjp < 10^–08^) and *mpz* mRNAs (FC = 7.08; adjp < 10^–03^) were alternatively spliced upon injury. These two genes code for components of the myelin sheath (Inouye and Kirschner, 2016). mRNAs encoded by *col12a1a* (FC = 4.5; adjp < 0.05), *mcamb* (FC = 3.59; adjp < 0.05), and *myo9aa* (FC = 3.6; adjp < 0.05) genes were also alternatively spliced after injury. These three protein-coding genes were associated with the development and the regeneration of axons (GO term). Transcripts synthesized from the transcription factor gene *nfia* were alternatively spliced comparing uninjured and injured telencephalic hemispheres (FC = 2.24; adjp < 0.05). The chicken homolog of *nfia* was implicated in the regulation of gliogenesis in the central nervous system (Kang et al., 2012). In response to telencephalon injury, the level of *nfia* transcripts decreased (FC = 1.25; adjp < 0.05), as well as levels of transcripts of its partners *sox9a* (FC = 1.20; adjp < 10^–03^) and *sox9b* (FC = 1.60; adjp < 10^–17^) (Supplementary Table 1).

**FIGURE 7:**
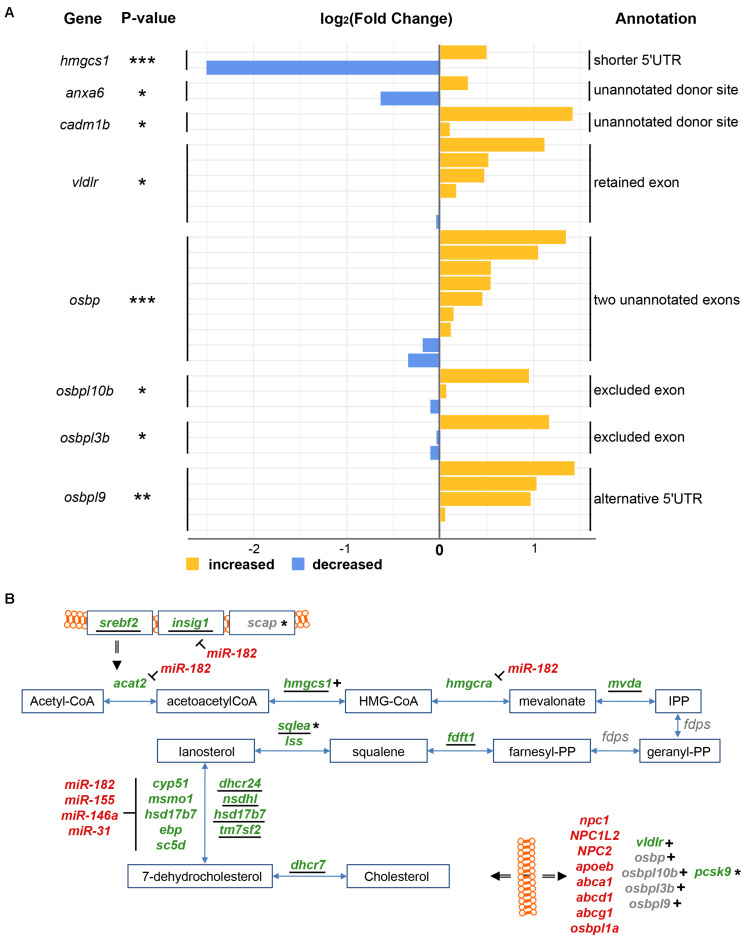
Alternative splicing of RNAs related to cholesterol metabolism in response to injury. **(A)** Splicing isoforms of RNAs encoding proteins of the cholesterol synthesis and transport pathway were first reconstructed and then quantified in both uninjured and injured telencephalic hemispheres. The color blue depicts a decrease in the number of supporting reads while yellow an increase. A number of splice isoforms were not yet annotated in the genome (unannotated). *adjp = 0.05, **adjp < 10^–02^, ***adjp < 10^–03^. **(B)** All results about cholesterol metabolism were finally integrated, including up-regulated transporters (red) down-regulated synthesizing enzymes (green) and genes encoding mRNA affected by alternative splicing (indicated by +) or predicted targets of microRNA or lncRNA (indicated by *). Underlined names depict genes harboring the SRE motif in their 1-kbp promoter.

We next focused specifically on the splicing patterns of genes involved in cholesterol metabolism (see Supplementary Figure for structures of spliced isoforms). In response to injury, the levels of mRNAs encoding the two related zebrafish splicing factors Ptbp1a and Ptbp1b significantly increased (FC = 1.57 and 1.20, respectively, adjp < 10^–08^ and < 0.05, respectively) (Supplementary Table 1). At the same time, the level of *hmgcs1* and *pcsk9* mRNAs decreased (FC = 1.60, 0.66, and 0.59, respectively, adjp < 10^–06^, < 10^–06^, and < 10^–04^, respectively) (Supplementary Table 1). These three genes are all involved in cholesterol metabolism in mammals (Sawicka et al., 2008; Medina and Krauss, 2013) and *hmgcs1* mRNAs were alternatively spliced in response to injury (adjp < 10^–04^) (Figure 7A). A decrease in the number of mapped reads spanning the longest 5‘UTR (ENSDARE00001157036, FC = 1.41) was consistent with a significant increase in the number of reads spanning the shortest 5′UTR (ENSDARE00001149813, FC = 0.18). Given that in human liver cells PTBP1 splices mRNA encoding HMGCS1 (Sawicka et al., 2008; Medina and Krauss, 2013), Ptbp1a/b are hypothetically involved in the splicing of the 5′UTR of *hmgcs1* transcripts in response to injury. This likely results in unstable isoforms thus contributing to the reduction of *hmgcs1* mRNA levels in the injured telencephalon.

mRNAs encoding proteins involved in cholesterol transport were also alternatively spliced after injury (Figure 7A). mRNAs encoding the Very Low Density Lipoprotein Receptor *vldlr* (adjp < 0.05) were spliced to exclude an exon (ENSDARE00001166020). No specific protein domain/function was annotated to this exon (InterPro) (Mitchell et al., 2019). VLDLs are responsible for extracellular cholesterol transport through the blood stream (Khosravi et al., 2018). Interestingly in contrast to all other cholesterol transporters, the overall level of *vldlr* transcripts significantly decreased upon injury (FC = 1.12; adjp < 0.05). Two non-annotated splice sites were discovered in exons of *anxa6* (ENSDARE00000906781, FC = 0.64 and 1.23, adjp < 0.05) and *cadm1b* (ENSDARE00000873208, FC = 1.07 and 2.66, adjp < 0.05). Anxa6 participates together with NPC proteins in the endosomal trafficking of cholesterol (Cubells et al., 2007), and Cadm1b has a predicted cholesterol 24-hydroxylase activity (GO term).

A total of four mRNAs encoding transporters of cholesterol metabolites of the OxySterol Binding (OSB) family (Yan et al., 2007) were also affected by splicing in response to telencephalon injury (Figure 7A). Two unannotated exons of *osbp* were discovered as newly emerging upon injury (adjp < 10^–05^). In response to injury, an exon was retained in mRNAs encoding *osbpl10b* (ENSDARE00000815047, adjp < 0.05) and *osbpl3b* (ENDARE00001041526, adjp < 0.05). No corresponding protein domain was annotated (InterPro). Two isoforms of mRNAs encoding Osbpl9b were alternatively spliced in response to injury, including an alternative 5’UTR (ENSDARE00000991106, adjp < 0.01) and a retained exon (ENSDARE00001127062, adjp < 0.01).

Taken together, our analysis identified alternative splicing as an important response to damage of the zebrafish telencephalon, suggesting distinct isoforms of proteins involved in the repair of the damaged tissue. The *de novo* reconstruction of the transcriptome also revealed novel isoforms of RNAs that emerged in response to injury of the telencephalon. Moreover, our data suggest that the mRNAs of cholesterol synthesizing and transporting proteins are subject to differential splicing thus contributing to the presumed adaptation of the cholesterol metabolism to the conditions in the injured brain (Figure 7B).

## Discussion

Unlike adult humans and other mammals, the adult zebrafish is able to efficiently repair injuries of the central nervous system. We analyzed here the transcriptome for changes in the expression of mRNAs, their splice variants and regulatory RNAs including analysis of the targets of regulated miRNAs and transcription factors in response to injuries of the telencephalon. We noted profound changes in genes belonging to a large number of distinct cellular and physiological processes. As exemplified by the coordinated regulation of the cholesterol synthesizing enzymes and transporters, the genome responded in a multi-tiered manner with distinct and interwoven changes in expression of regulatory molecules to the physiological demands created by tissue damage and its repair. This multi-level regulation of the expression of cholesterol metabolizing proteins uncovers an important process in the regenerating telencephalon. Our comprehensive analysis provides moreover an important source of information for future in-depth functional studies of specific genes and gene groups, regulatory molecules and splice variants in the regenerating zebrafish forebrain.

### Large Scale Response of the Genome to Telencephalon Injury

The analysis of our sequencing data (Rodriguez-Viales et al., 2015) with more than 600,000,000 reads from polyadenylated RNAs of control and injured telencephala revealed a change in expression of 4,989 genes. This represents 15% of all genes analyzed and 29% of all genes detectably expressed in the samples. Thus, injury causes profound changes in the expression of information from the genome.

Regeneration of the adult zebrafish telencephalon is a complex process that entails many distinct physiological changes such as immune response, activation of glial cells, proliferation of stem cells, neurogenesis, axonogenesis etc. (Schmidt et al., 2013). These previously described processes were all detected in our gene ontology analysis of protein coding genes adding an independent verification of our data and their analysis. In total, we scored 521 gene ontology terms and pathways with significant overrepresentation (adjp < 0.05) in the transcriptome of the injured telencephalon relative to the uninjured control. These findings are in agreement with the large scale and complex demands of new proteins to cope with the inflicted injury. We had prepared cDNA from tissues at 5 dpl. At this time, the peak of proliferation of stem cells is reached (Rodriguez-Viales et al., 2015). Thus, the changes not only entail immediate early reactions to damage such as immune reaction but also genes with functions in repair of tissue function such as neurogenesis and axonogenesis.

Our findings on mRNA expression profiles are complementary to a recently published transcriptome study focusing on the immediate early changes in response to injury of the telencephalon (Demirci et al., 2020). Interestingly, this study reported activation of gene expression programs in both the injured and the uninjured hemispheres, even though the response was less pronounced and delayed in the injured hemisphere. In contrast, we never observed proliferation of stem cells or stem cell gene activation in the unlesioned hemisphere (März et al., 2010b). We systematically analyzed hundreds of transcription regulators for their expression in the injured telencephalon by *in situ* hybridization on sections. We did not observe gene activation in the uninjured hemisphere (Diotel et al., 2015; Rodriguez-Viales et al., 2015; Demirci et al., 2020). Demirci et al. (2020) inflicted lesions by inserting a needle into the nostril. In contrast, our protocol of injuring the telencephalon (Schmidt et al., 2014) involves the inserting the needle directly into one hemisphere of the telencephalon. Most likely the protocol used by Demirci et al. (2020) causes damage of the second hemisphere or some of the extending nerves thereby causing activation of regenerative programs also in the seemingly uninjured hemisphere.

### Profound Changes in Splicing Patterns in Response to Injury

The term “mRNA splice site selection” was also enriched among the genes with altered expression in the injured brain,–with 8 genes down-regulated in response to injury. This observation is in agreement with our systematic analysis of splice variants. We detected changes of splice patterns in 4,610 transcripts representing 1,309 genes. Thus, not only the overall levels of mRNAs were adapted to the physiological demands imposed by injury and repair but also the posttranscriptional processing of the mRNAs. In support, alternative splicing was reported for the modulation of the function of specific genes during neurogenesis in mammals (Su et al., 2018; Lee et al., 2020). For example, in the developing mouse brain, the splicing factor PTBP2 targets mRNAs encoding DNM1 and modulates synaptic vesicle trafficking (Li et al., 2014). In the zebrafish, to our knowledges, no comprehensive study investigated alternative splicing of mRNAs in the CNS. Deficiency in Rnpc3 splicing factor results in multiple impairments during development of zebrafish embryos (Markmiller et al., 2014). Also Neuro-Oncological Ventral Antigen 1 and 2 are splicing factors required for the correct development of the zebrafish brain (Jelen et al., 2007). The mRNA isoforms were in most cases detected in both uninjured and injured telencephalic hemispheres. This suggests that injury causes a modulation of the function by shifting from one isoform to the other. Alternative splicing of mRNAs can also lead to the degradation of mRNAs (Lareau et al., 2007). Thus, alternatively, this shift of the predominant splice isoforms could thus be a means for adjusting the expression levels to the new physiological needs in the injured brain.

Taken together, our data suggest that alternative splicing represents another major response of the genome to cope with the physiological demands of the regenerating telencephalon. Since all splice variants were expressed in transcriptomes of controls and injured telencephala albeit at different levels, alternative splicing does not seem to control all-or-none effects but appears to be rather involved in the fine-tuning of the expression levels or functions of constitutively expressed genes.

### Alteration in Cholesterol Metabolism in Response to Telencephalon Injury

“Cholesterol biosynthesis” is a prominent gene ontology term among the genes whose expression was altered in response to injury. Cholesterol synthesis involves a pathway that initiates with the multistep synthesis of lanosterol from acetyl-CoA as precursor. Lanosterol is then converted by a whole battery of enzymes into 7-dehydrocholesterol and ultimately into cholesterol. With the exception of *fdps* mRNA, the mRNAs encoding cholesterol synthesizing enzymes of each of the steps from acetyl-CoA to cholesterol are down-regulated in the injured telencephalon. This suggests that cholesterol synthesis is co-ordinately reduced in response to injury. Our results are supported by recent studies in rat and mouse. As, for example, the inhibition of cholesterol synthesis by statins results in an increase in the number of neurons and retinal ganglion cells reforming the optic nerve after injury (Shabanzadeh et al., 2021). In the zebrafish hindbrain, activation of SREBP promotes the myelination of neurons (Ashikawa et al., 2016).

Intriguingly, mRNAs encoding cholesterol transporters are elevated in the injured telencephalon. Transporters include also proteins associated with transport across endosomal membranes (Cubells et al., 2007) suggesting alteration of cholesterol fluxes both across the plasma membrane and also within the cell into the endosomal compartment. A recent study in the cerebellum in NPC1 deficient mouse reports an increase in cholesterol storage in microglial cells and impairment in myelination of neurons (Colombo et al., 2021). Another mouse model, deficient in ApoE, shows impaired formation of dendrites in injured adult hippocampus (Tensaouti et al., 2020). These studies suggest that storage of cholesterol and rebuilding of the injured tissue are tightly linked.

There is also a link between cholesterol metabolism and the inflammatory response. The transcription factor Liver-x-receptor regulates cholesterol metabolism and the inflammatory response (Bilotta et al., 2020). Moreover, the sterol metabolite 25-hydroxycholesterol modulates the inflammatory response (Gold et al., 2014). In light of the immune response being an important trigger of neurogenesis in the adult zebrafish telencephalon (Kyritsis et al., 2012), the observed expression changes may promote an immune response and thus regeneration.

Taken together, the regenerating telencephalon thus appears to systematically reprogram cholesterol metabolism from synthesis to relocation of cholesterol with three hypothetical purposes: (i) Provision of material for remyelination of damaged neurons, (ii) Efficient clearance of cell debris, (iii) Activation and the maintenance of the immune response.

### Putative Regulation of Cholesterol Synthesizing Enzymes by Srebf2

In mammals, cholesterol synthesis is tightly regulated by posttranscriptional mechanisms involving the retention of the SREBF transcription factor in the ER (Wang et al., 1994). At high levels of available cholesterol, Srebf2 is associated with Insig1 and Scap at the membranes of the endoplasmic reticulum (ER) and Golgi apparatus. Upon cholesterol shortage, this repressive association is dissolved and Srebf2 moves to the nucleus where it binds to the promoters of genes encoding the various enzymes of the cholesterol synthesis pathway and thereby induces the expression of the enzymes. In mammalian genomes, there are two related *Srebf* genes, *Srebf1*, and *Srebf2*, with Srebf2 being predominantly involved in regulation of genes encoding cholesterol synthesizing enzymes (Wang et al., 1994; Eberlé et al., 2004; Sharpe and Brown, 2013). Similarly, the zebrafish genome harbors two *srebf* genes highly related to mammalian *srebf1* and *srebf2*.

According to previous (AGETAZ database; Diotel et al., 2015) and current results, both Srebf1 and -2 are expressed in the adult zebrafish telencephalon. Our bioinformatic analysis of the 1-kb promoter upstream regions of genes encoding cholesterol synthesizing enzymes in the zebrafish genome revealed a strong enrichment of Srebf binding sites. Also *insig1* and *scap* mRNAs are expressed in the zebrafish telencephalon and level of *insig1* mRNA decreased upon injury.

Our comparative analysis of the injured and uninjured telencephalic hemisphere uncovered, however, in addition regulation of the *srebf2* mRNA level: *srebf2* mRNA was less abundant in the injured telencephalic hemisphere in agreement with the decreased expression of cholesterol synthesizing enzymes. Intriguingly, we detected a Srebf2 binding site in the promoter of the *srebf2* gene suggesting auto-regulation via a positive feedback loop. Taken together, our *in silico* analysis suggests that the regulation of the level of *srebf2* mRNA is a potential mechanism how cholesterol synthesis is adjusted to the needs of the regenerating zebrafish telencephalon. In view of the expression of the Srebf2 regulators *insig1* and *scap* in the adult zebrafish telencephalon, it is, however, likely that Srebf2 activity is regulated by the canonical posttranscriptional mechanism in addition to abundance of the Srebf2 protein.

### MicroRNAs as Additional Regulatory Mechanisms of Cholesterol Metabolism in the Injured Telencephalon

miRNAs are well known as regulators of large gene batteries (Barca-Mayo and De PietriTonelli, 2014). A total of 184 miRNAs annotated in the zebrafish reference genome (GRCz11) were detectable by small RNASeq in the transcriptome of the adult zebrafish telencephalon. Of these, 31 miRNAs varied in level of expression upon injury. These miRNAs are distinct from miRNAs implicated previously in constitutive neurogenesis (Rajman and Schratt, 2017) and regeneration of the zebrafish optic nerve (Fuller-Carter et al., 2015). Thus, the scale and type of damage of the telencephalon may trigger specific responses both with respect to clearance of dead tissue, neurogenesis and regenerative processes. Given the fact that we prepared small RNAs from entire injured and uninjured hemispheres, it cannot be totally excluded that we failed to detect changes in miRNA expression in low-abundant cells such as stem cells and neuroblasts (März et al., 2011). However, we detected constitutive expression of miR-9 which is expressed in neural stem cells in the telencephalon (Coolen et al., 2013). Our sensitivity of detection appears thus high and includes also stem-cell-specific miRNAs.

Potential targets of miRNAs were identified by the presence of the binding site of miRNAs in the 3’UTR of mRNAs expressed in the injured and uninjured telencephalic hemisphere. Intriguingly, the expression of the miRNAs, *miR-182*, *miR-31*, *miR-155*, and *miR-146a*, which were most strongly up-regulated in response to injury are all linked to the regulation of cholesterol synthesis. The *miR-182* seed sequence was found in the mRNAs encoding the enzymes Acat2, Hmgcs1, and Dhcr24 of the cholesterol synthesis pathway. The level of these mRNA was consistently decreased upon injury. *miR-182* targets also mRNAs coding for the Srebf2 co-regulator Insig1. Thus, *miR-182* appears to affect cholesterol metabolism at two levels: (i) The regulator Insig1 (ii) Selected synthesizing enzymes. The seed sequences of *miR-31*, *miR-155*, and *miR-146a* were present in the 3′UTR of five mRNAs coding for enzymes of the conversion of lanosterol into 7-dehydrocholesterol, also with consistent decrease in their respective levels. In the mouse liver, depletion of *miR-155* resulted in an increase in hepatic level of cholesterol (Miller et al., 2013). Similarly, *miR-146a* was shown to regulate the plasma level of cholesterol (Del Monte et al., 2018). These observations in mice are consistent with the inferred role of these miRNAs regulating cholesterol synthesis in the injured zebrafish telencephalon.

In summary, these miRNAs could provide additional regulatory inputs that act in parallel with the Srebf2 factor on cholesterol synthesizing enzymes. Curiously, the three miRNAs, *miR-31*, *miR-155*, and *miR-146a* target all one section of the cholesterol synthesis pathway, the conversion of lanosterol into 7-dehydrocholesterol (Figure 3A). This suggests that these may be key steps that need tight control.

### Alternative Splicing as an Additional Mode of Regulation of Cholesterol Metabolism

Alternative splicing that can increase the diversity of proteins (Choi et al., 1980) or leads to degradation of mRNAs or proteins (Bartel, 2004) was noted for a number of genes involved both in synthesis and transport of cholesterol. In mammals, polypyrimidine tract binding protein 1 (PTBP1) splices mRNAs encoding several proteins of the cholesterol metabolism including the enzymes HMGCS1, and PCSK9 (Medina et al., 2011; Medina and Krauss, 2013). We found that the levels of mRNAs encoding the two zebrafish homolog Ptbp1a and Ptbp1b were significantly increased suggesting a role of Ptbp1a/b proteins in the regulation of cholesterol metabolism in the injured telencephalon of the zebrafish. In agreement, we observed a shift in the splice patterns of the zebrafish homolog of *hmgcs1* mRNA in response to injury. In mammals, this shift in splice patterns of *hmgcs1* was paralleled by an overall decrease of the three proteins (Medina and Krauss, 2013). This suggests that the action of increased *ptp1a/b* leads to isoforms that are less stable, thereby contributing to the systemic decrease in the expression of mRNAs encoding cholesterol synthesizing enzymes in the injured telencephalon. Together with the known role of PTBP2 in splicing of mRNAs encoding proteins of synaptic vesicle trafficking in the developing mouse brain (Li et al., 2014), our results strengthen a link between regulation of cholesterol efflux and functional repair of damaged adult zebrafish telencephalon.

Another protein with alternatively spliced mRNA is Cadm1b that has a predicted Cholesterol 24-hydroxylase activity (GO term). Cholesterol is esterified to 24-hydroxycholesterol to regulate cholesterol homeostasis via storage (Zhang and Liu, 2015). Interestingly we also found that the promoter of *cyp39a1* harbors a SRE binding motif and that the level of *cyp39a1* mRNA decreases upon injury. Cyp39a1 is a monooxygenase converting 24-hydroxycholesterol into precursor of steroids and other lipids (Li-Hawkins et al., 2000). These results suggest that downstream usage of cholesterol, as precursor, is limited to the benefits of its storage after injury of the telencephalon.

Several mRNAs encoding transporters of cholesterol were also alternatively spliced after injury. These include mRNAs of the Very Low Density Lipoprotein (LDL) Receptor *vldlr* which binds LDLs responsible for cholesterol transport through the blood stream (Khosravi et al., 2018). Together with ApoE receptor, VLDL receptor participates in brain development modulating the Reelin signaling pathway (Lane-Donovan and Herz, 2017). This latter is critical for synaptic formation and plasticity as well as morphogenesis of mouse cerebellum (Jossin, 2020). Anxa6 together with NPC proteins mediates endosomal trafficking of cholesterol (Cubells et al., 2007). Several transporter of the OSB family (Yan et al., 2007) were affected by alternative splicing. None of the alternatively spliced amino acid coding exons has a specific annotated function or structure in the InterPro database (Mitchell et al., 2019). It remains thus to be seen whether these alternatively spliced proteins have altered properties such as function, stability or subcellular locations. OSB proteins bind cholesterol and cholesterol derivatives, as 24- and 25-hydroxycholesterol, participating in the intracellular homeostasis of cholesterol by facilitating trafficking between the organelles (Raychaudhuri and Prinz, 2010). As cholesterol exerts a regulation of its own synthesis, OSB proteins also indirectly participate in the regulation of cholesterol synthesis. OSB proteins are also known to sense cholesterol and to mediate downstream cell signaling pathways such as JAK/STAT and ERK.

Taken together our results suggest that alternative splicing is an important process contributing to the altered expression/activity of cholesterol metabolizing proteins in response to injury.

### Relevance of Modulation of Cholesterol Metabolism

Key questions are why cholesterol metabolism is so tightly regulated and why the down-regulation of it is a feature of the injured telencephalon. Cholesterol is an essential component of many cellular processes. It determines the biophysical and biochemical properties of membranes. It is a precursor of steroid hormones, and cholesterol derivatives are important secondary modifications of proteins such as Wnt receptors (Sheng et al., 2014) or Hedgehog ligands (Purohit et al., 2020). Thus and given its general hydrophobic property, free excess cholesterol has an impact on the functional integrity of membranes and the communication between cells. Debris from damaged axonal processes, in particular their membrane-rich myelin sheaths, may likely be abundant sources of extracellular cholesterol. It is probably very critical for cell survival and efficient repair of the brain to control free cholesterol levels very tightly. Moreover, as discussed above cholesterol may be involved as a proinflammatory cue to drive regenerative neurogenesis as well as myelination.

The coordinated down-regulation of expression of genes encoding cholesterol synthesizing enzymes and the up-regulation of transporters suggests that the injured telencephalon switches from synthesis of cholesterol to its import from the extracellular environment. In addition, the up-regulation of genes coding for cholesterol transporters (Npc1, Npc2), which are linked to endosomes (Cubells et al., 2007; Colombo et al., 2021) may reflect an additional switch to cholesterol storage in microglial cells or excretion rather than synthesis. In this context, it is important to note that the blood brain barrier prevents efficient exchange of cholesterol between the brain and the rest of the body (Björkhem and Meaney, 2004), possibly necessitating this tight control within the brain. In the injured brain of mice (Wong et al., 2020), cholesterol 25-hydroxylase levels are increased in microglial cells. This enzyme converts cholesterol into a more hydrophilic form and allows there by its crossing of the blood brain barrier, metabolism in the liver and excretion via the bile. We observed an elevated level of cholesterol 25-hydroxylase mRNA in the injured zebrafish brain suggesting that increased efflux to the liver may also be a mechanism to reduce cholesterol levels in the injured zebrafish brain. Furthermore, in the injured mouse and rat brain, APOE, a transporter of cholesterol, is increased upon injury (Castranio et al., 2018; Chong et al., 2019). Thus, also in the mouse, cholesterol metabolism and transport appears to be increased upon injury.

However transport to the liver may not be the only important transport route. Genes involved in cholesterol transport into vesicles for purposes of storage within the brain are also activated. These brain intrinsic stores may be instrumental for the ordered reassembly of damaged brain structures. In agreement, mice deficient in ApoE present impaired formation of dendrites (Tensaouti et al., 2020).

Despite several anecdotal lines of support in the literature, to our knowledge, the systemic regulation of cholesterol metabolizing enzymes which we observed in the injured zebrafish telencephalon was not reported so far for the mammalian brain. This control of cholesterol metabolism may be of medical relevance. It may open possibilities to combat the outcomes of conditions like stroke, injury and neurodegeneration in the human brain. In support, decreasing the level of circulating cholesterol in the rat improves the recovery after brain injury (Chong et al., 2019). Taken together, our comparative *in silico* analysis of the transcriptomes of the injured and uninjured telencephalon of the adult zebrafish suggests that regulation of cholesterol levels is an important process for brain regeneration.

### Multi-Layer Regulation of Cholesterol Metabolism a Means of Robustness or a Mood of Evolution?

We showed here that, cholesterol metabolizing genes appear to be regulated at multiple levels in response to injury of the telencephalon. At the level of inferred protein functions, an overall switch from synthesis to transport and possibly also storage and metabolism/excretion of cholesterol is evident from the comparative analysis of the transcriptomes of the injured and uninjured telencephalon. When the putative regulatory mechanisms were explored, changes of expression of regulatory molecules suggested multiple synergistic and complementary regulatory networks controlling cholesterol synthesis and transport. These include a decrease of Srebf2 mRNA leading to reduction of the key transcription activator of most enzymes of the cholesterol synthesis pathway. Drawing from the mammalian literature (Ulitsky et al., 2012), the expression of key regulators (Insig1, Scap) in the zebrafish telencephalon and given the general high conservation of many regulatory mechanisms between fish and mammals, it is likely that the activity of zebrafish Srebf2 is also regulated by subcellular distribution. Thus, in addition to down-regulation of the *srebf2* mRNA, the corresponding protein is likely not located in the nucleus in the injured telencephalon. Changes in splice patterns of cholesterol synthesizing enzymes and transporters may alter protein function or lead to degradation of the mRNA or encoded enzymes adding another principle of regulation. Further layers of regulation are conferred by changes in expression of regulatory RNAs. Up-regulation of miRNAs targeting the mRNAs of a subgroup of cholesterol synthesizing enzymes contributes to the decrease of the target RNAs. Changed expression of lncRNAs at the loci of several genes encoding cholesterol synthesizing or transporting proteins offer yet other layers of regulatory principle woven into the control of cholesterol metabolism.

A key question is why cholesterol metabolism requires such a complex multi-layered control. The transcriptional changes in cholesterol metabolizing genes and their multilevel regulation may be a reflection of the brain’s autonomy with respect to cholesterol metabolism. The crucial biological functions of cholesterol and the pathogenic effects of excessively high cholesterol levels may call for efficient and robust mechanisms. This robustness may be best achieved by complementary and synergistic modes of regulation. Alternatively, this architecture of regulatory mechanisms may be a reflection of how living systems evolve. By randomly recruiting and adapting components of the cells existing repertoire of gene regulatory mechanisms, this seemingly rather complex regulatory network architecture may have arisen. As the evolved mechanisms were effective, they were maintained. Thus, this complexity most likely reflects both evolutionary process and robustness in adaptation of cholesterol levels to the physiological state during injury and repair of the brain.

## Data Availability Statement

mRNAseq and small RNAseq data are available on the Gene Expression Omnibus data base under the accession identifiers GSE161137 and GSE160992, respectively.

## Ethics Statement

The animal study was reviewed and approved by the Government of the Baden-Württemberg, Regierungspräsidium Karlsruhe, Germany.

## Author Contributions

US, OA, and SR designed the study. OA carried out the sequencing experiments. LL and ND tested the reproducibility of the results. VG analyzed and integrated the results. US, VG, and OA interpreted the results. US and VG wrote the manuscript. All authors read and approved the manuscript.

## Conflict of Interest

The authors declare that the research was conducted in the absence of any commercial or financial relationships that could be construed as a potential conflict of interest.

## Footnotes

1 https://m.ensembl.org/info/genome/genebuild/ncrna.html

2 http://hannonlab.cshl.edu/fastx_toolkit/index.html

## Supplementary Material

Supplementary Figure 1 Reconstruction of alternatively spliced isoforms of transcripts related to cholesterol metabolism. Solid square: annotated exon; dashed square: novel exon; red: increased usage of junction; green: decreased usage of junction; 5′: 5′UTR; ^∗^: stop codon; number: Ensembl exon identifier.

Supplementary Table 1 Polyadenylated RNAs with significantly changed levels upon injury.

Supplementary Table 2 Significantly enriched biological functions among differentially expressed genes.

Supplementary Table 3 Differentially expressed genes encoding proteins with function related to cholesterol metabolism.

Supplementary Table 4 Comparison of genes with cholesterol biosynthetic function in the human and zebrafish genome and with mapped SRE motifs in the 1-kb promoter.

Supplementary Table 5 SRE motifs mapped in the promoter of genes with functions related to cholesterol biosynthesis in the zebrafish genome.

Supplementary Table 6 Genes differentially expressed in response to injury with SRE motif(s) in the 1-kb promoter region.

Supplementary Table 7 microRNAs with significant changes in level upon injury.

Supplementary Table 8 Significant alternative splicing in response to injury.

Supplementary Table 9 Significantly enriched biological functions among differentially expressed genes harboring the SRE motif in their promoters.

Supplementary Table 10 Sequence of the primers for the validation by qRT-PCR.

## Abbreviations

adjp: adjusted *p*-value
cDNA: complementary deoxyribonucleic acid
ChIP: chromatin immuno-precipitation
CNS: central nervous system
dpl: day post lesion
ER: endoplasmic reticulum
FC: fold change
FDR: false discovery rate
GO: gene ontology
hpl: hour post lesion
kb: kilobase
KEGG: Kyoto encyclopedia of genes and genomes
LDL: low density lipoprotein
mRNA: messenger RNA
miRNA: microRNA
lncRNA: long non-coding RNA
NSC: neuronal stem cell
OSB: oxy sterol binding
qRT-PCR: quantitative reverse transcription polymerase chain reaction
RGC: radial glial cell
RNA: ribonucleic acid
SRE: sterol regulatory element
SREBF: sterol regulatory element binding family
TF: transcription factor
UTR: untranslated region.
