# Supplementary figures and images for: Multi-Dimensional Transcriptome Analysis Reveals Modulation of Cholesterol Metabolism as Highly Integrated Response to Brain Injury
Source: Front Neurosci. 2021 May 14;15:671249. doi: 10.3389/fnins.2021.671249 (PMC8162057; doi:10.3389/fnins.2021.671249)

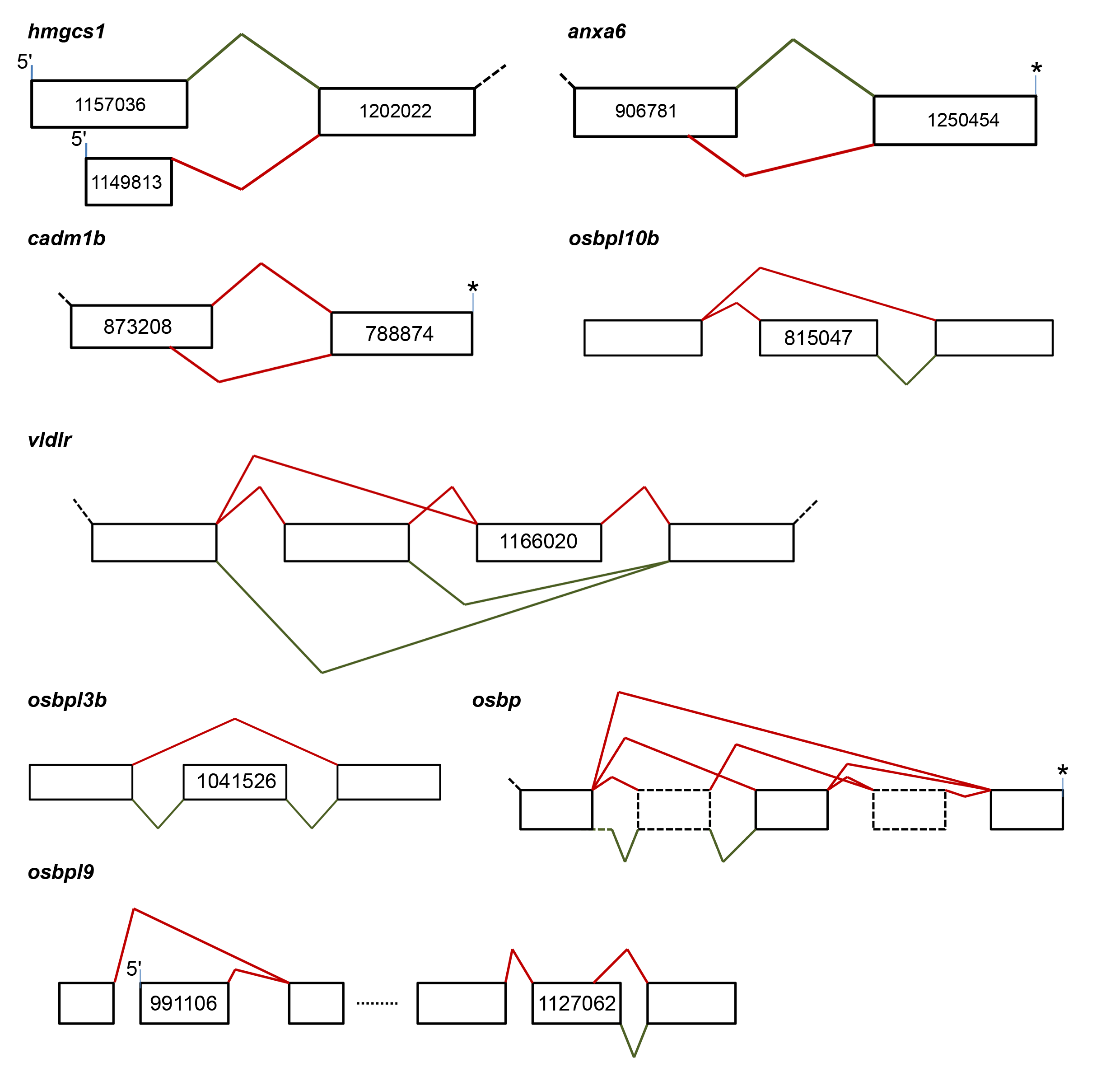

Supplement: Supplementary Figure 1 — Reconstruction of alternatively spliced isoforms of transcripts related to cholesterol metabolism. Solid square: annotated exon; dashed square: novel exon; red: increased usage of junction; green: decreased usage of junction; 5′: 5′UTR; ∗: stop codon; number: Ensembl exon identifier. [file Image_1.TIF]
